# Proton induced DNA double strand breaks at the Bragg peak: Evidence of enhanced LET effect

**DOI:** 10.3389/fonc.2022.930393

**Published:** 2022-08-05

**Authors:** Cara M. Frame, Yu Chen, Jonathan Gagnon, Y. Yuan, Tianjun Ma, Anatoly Dritschilo, Dalong Pang

**Affiliations:** Department of Radiation Medicine, Georgetown University Hospital, Washington, DC, United States

**Keywords:** AFM, proton, Bragg peak, DNA, DSB

## Abstract

**Purpose:**

To investigate DNA double strand breaks (DSBs) induced by therapeutic proton beams in plateau and Bragg peak to demonstrate DSB induction due to the higher LET in the Bragg peak.

**Materials and Methods:**

pUC19 plasmid DNA samples were irradiated to doses of 1000 and 3000 Gy on a Mevion S250i proton system with a monoenergetic, 110 MeV, proton beam at depths of 2 and 9.4 cm, corresponding to a position on the plateau and distal Bragg peak of the beam, respectively. The irradiated DNA samples were imaged by atomic force microscopy for visualization of individual DNA molecules, either broken or intact, and quantification of the DNA fragment length distributions for each of the irradiated samples. Percentage of the broken DNA and average number of DSBs per DNA molecule were obtained.

**Results:**

Compared to irradiation effects in the plateau region, DNA irradiated at the Bragg peak sustained more breakage at the same dose, yielding more short DNA fragments and higher numbers of DSB per DNA molecule.

**Conclusion:**

The higher LET of proton beams at the Bragg peak results in more densely distributed DNA DSBs, which supports an underlying mechanism for the increased cell killing by protons at the Bragg peak.

## Introduction

The development of dedicated clinical proton technology has accelerated proton radiotherapy adoption and renewed interest in understanding the fundamental physical and biological properties of proton interactions with living tissues and cells. The RBE variation along the proton Bragg peak, especially towards the distal falloff of the peak has been of particular interest and clinical relevance (1–4). Practical considerations have led to the adoption of an average value of 1.1 for the biological effectiveness of protons relative to photons, regardless of proton energy or depth of beam penetration. However, recent *in vitro* studies of cell survival have demonstrated a continuous increase in RBE along a proton path and a rapid increase in RBE in the region of the Bragg peak, reaching the highest value of 1.7 (5–8). Furthermore, reported clinical observations have been interpreted to support a non-constant RBE (9, 10). Unexpected brainstem injuries of patients receiving proton brain radiation have been attributed to the effect of increased RBE at the distal end of the proton beam (11, 12). Based on laboratory and pre-clinical data, models have been formulated for calculation of proton RBE as a function of dose, energy and depth (13, 14), and the RBE variations have been incorporated into radiation dose distribution calculations (15).

In addition to cell survival studies, RBE effects have also been investigated by measurements of proton induced DNA DSB. Belli etal. (16) measured DSB in V79 cells by low energy protons using low speed sedimentation technique. They observed a linear correlation between DSB yield and dose but not with LET. Campa etal. (17) measured DSB induced by 0.84 MeV, low energy protons using gel electrophoresis and also reported a linear response with dose. More recently Chaudhary etal. (18) investigated DNA damage and repair along a 60 MeV proton beam path using the 53BP1 foci formation assay and found significant persistence of foci at the distal end of the proton SOBP, suggesting more complex DNA DSB induction by the higher-LET protons. Cuaron etal. (19) and Keta etal. (20) also studied DNA damage using the γH2AX assay by therapeutic proton beam in plateau and distal edge of the SOBP, and found persistently higher γH2AX signal at the distal edge.

Using a plasmid model system, Vyšín etal. (21) studied both direct and indirect damage of DNA by low energy proton irradiation using the agarose gel electrophoresis assay. Using a mathematic model to fit the electrophoresis profile, they derived the number of DSBs per Mbp. The DNA samples were placed in the plateau region and therefore no comparisons were made between the effect of Bragg peak and that of the plateau.

Atomic Force Microscopy (AFM) is an established imaging technique capable of atomic resolution for solid state materials and nanometer resolution for soft biomolecules (22). AFM has been used to image individual DNA molecules and DNA fragments following exposure to neutron, electron, and other ion irradiations for determination of DNA DSBs (23–25). In this investigation, we applied AFM imaging to quantify proton induced DNA breaks using the same plasmid DNA model system (pUC19) that we have used in previous studies at various positions along a proton beam. Compared to other biological assays for DNA strand break measurement, AFM offers the unique ability to measure individual, short DNA fragments of a few nanometers in length and it does not require mathematical models for calculation of DSBs, which can introduce biases due to assumptions in the model. As a result, this approach permits accurate, high-resolution quantification of densely distributed DSBs, and therefore is suited to measure DSBs by high-LET radiations. We demonstrate enhanced DNA breakage at the proton Bragg peak in comparison to the plateau regions at the same doses. These observations are interpreted as direct evidence of the increased LET effects on DNA DSB induction and are consistent with the reported RBE increase for cell killing at the Bragg peaks.

## Materials and methods

### a. DNA sample preparation

PUC19 plasmid DNA (purchased from New England Biolab in Beverley, MA) in its original concentration of 1 mg/ml in buffer (10 mM Tris-HCl, 1 mM EDTA) was diluted to 5 ng/µl in buffer consisting of 10 mM HEPES and 1 mM MgCl_2_.

### b. Proton irradiation

Proton irradiation of the pUC19 DNA was performed on a Mevion S250i proton system at the Proton Center of Medstar Georgetown University Hospital. The Mevion S250i is a compact, single room clinical proton system with highest energy of 227 MeV. A 50-ton super-conducting synchrocyclotron which is directly mounted on the treatment gantry produces proton beams to yield a dose rate of 2 Gy/min at iso center in a 10 cm^3^ water volume. Proton scanning is facilitated by a single magnet, dual coil beam scanning apparatus that permits scanning in X- and Y-direction at a scanning speed of 10 m/s. Energy modulation is accomplished with use of an energy selector consisting of 18 Lexan plates of various thicknesses. Insertion of plates of proper thicknesses into the beam reduces the 227 MeV proton beam from the accelerator to desired energies, ranging from 227 MeV to 0 MeV, permitting treatment from skin surface to a depth of 32 cm. The proton spots are further trimmed by a dynamic Adaptive Aperture on the field periphery to reduce beam penumbra. The entire beam monitoring and modulating devices are housed in the treatment nozzle of less than 2 m in length (26).

To facilitate irradiation of the DNA samples at specified depths along the path of proton beam, a 1-cm diameter and 1-mm depth well was drilled in a 2-cm thick, 30cm x 30cm solid water plate at its center. The well can hold 70 µl liquid. A single spot, 110 MeV proton beam was chosen to irradiate the liquid DNA samples at a water equivalent depth of 2 and 9.4 cm, respectively, corresponding to a point on the flat plateau and the distal 50% Bragg peak position. Selection of a single spot beam for irradiation reflects consideration of the proton system to produce high doses in the kGy range for our DNA DSB measurement technique (24). The sigma of the 110 MeV beam is 14.7 mm at the water surface using this setup, and gradually increases with depth in water to reach a maximum of roughly 18 mm at the Bragg peak position. When aligned to the center of the 10-mm diameter well, the 110 MeV single spot beam ensures dose uniformity of 93% in the DNA containing well, taking into consideration a potential 1-mm positioning uncertainty. In the depth direction, when positioned at the Bragg peak with a 1-mm positioning uncertainty, the dose variation in the 1 mm-deep well is less than 5% in the plateau and 10% in the distal Bragg peak.

Using an anterior beam and water equivalent solid water plates, the position of the DNA containing chamber on the 110 MeV beam path is determined by the thickness of the plates, which was calculated using the commercial Raystation treatment planning system and verified by dose measurements using a calibrated PPC05 parallel plate chamber. **Table 1** shows calculated doses at 2 cm depth on the plateau and at 9.4 cm depth at 50% distal Bragg peak. Also shown are the monitor units required to deliver 1000 Gy at the two positions and the corresponding LET values.

**Table 1:** Calculated doses and corresponding LETs at the two irradiation positions.

ROI	Depth (cm)	Physical Dose Rate (cGy/MU)	MU for 1000 Gy	LET (keV/µm)	Dose delivery uncertainty
Plateau	2.0	0.78	128,000	1.11	5%
Distal Bragg peak	9.4	0.95	106,000	22.60	10%

Measurements were performed for verification of the calculated doses. The last column shows the dose uncertainty of 5% and 10% at the two positions, respectively due to the maximum 1-mm position uncertainty.

### c. AFM imaging and measurement of DNA fragments

The irradiated DNA samples were extracted from the well using a 100 µl pipette and stored in Eppendorf tubes at –20^0^C until use. As a control, an unirradiated DNA sample was subjected to the same storage process. In preparation for AFM imaging, a 2 µl DNA containing buffer was deposited on freshly cleaved mica, followed by a gentle rinse with 600 µl distilled/deionized water, and dried in a gentle flow of nitrogen gas. The AFM images were obtained using a Bruker NanoScope-8 model, operated in ScanAsys mode at a scanning frequency of 1 Hz and scanning area of 2 x 2 µm^2^.

Measurements of fragment lengths were obtained using a commercial software package, the FemtoScan. Construction of DNA fragment length distributions, from which the number of DSB per DNA, the number of DSB per broken DNA and the spatial distribution of DSB on a DNA molecule follow procedures and formula previously established (24, 25)

## Results

**Figure 1:**
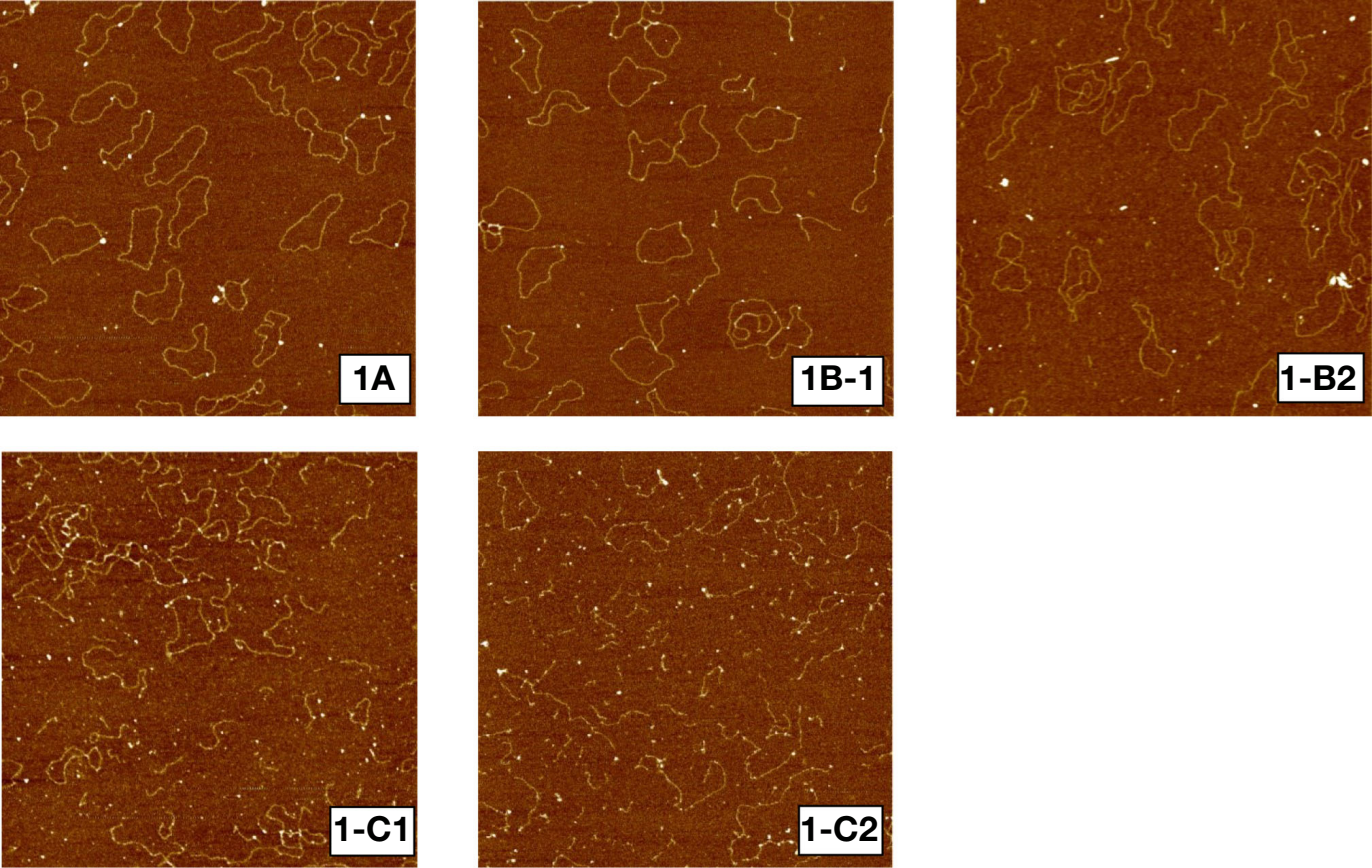
Sample AFM images of pUC19 plasmid DNA. 1a: unirradiated, 1b-1: irradiated to 1000 Gy at 2cm depth, 1b-2: irradiated to 1000 Gy at 9.4 cm depth, 1c-1: irradiated to 3000 Gy at 2 cm depth, 1c-2: irradiated to 3000 Gy at 9.4 cm depth.

**Figure 2:**
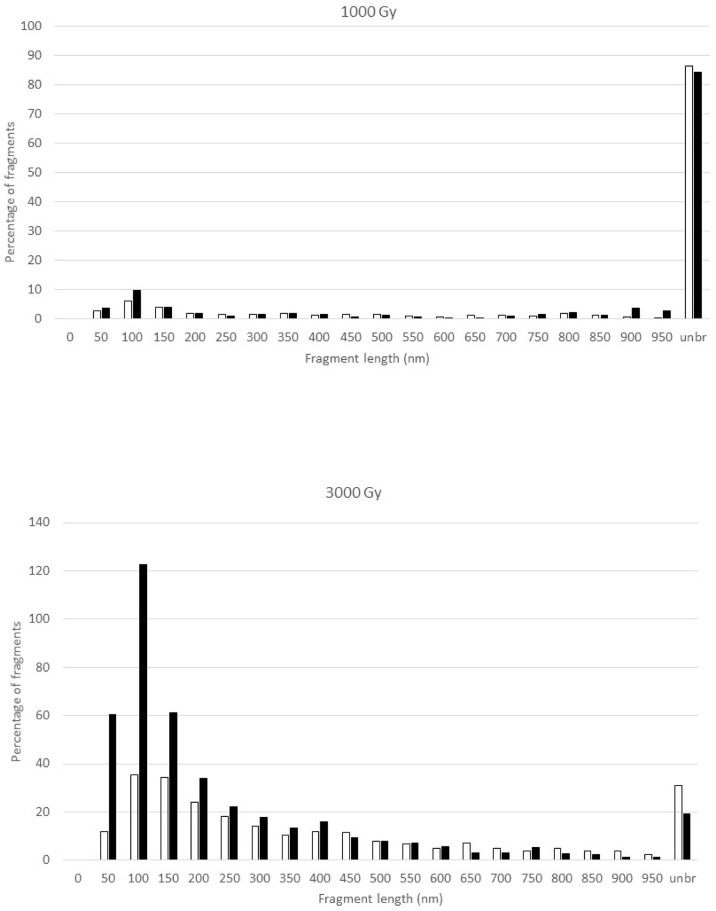
Fragment size distribution of the pUC19 plasmid DNA after exposure to 1000 Gy (upper panel) and 3000 Gy (lower panel) proton irradiation in the plateau (2-cm depth, open bar) and at the peak (9.4-cm depth, solid bar) of the 110 MeV Pristine proton bream. Where the number of fragments in each length bin is normalized to the total number of DNA molecules that include both broken DNA and intact DNA, which results in the percentage of fragments greater than 100 for the 3000 Gy irradiated DNA samples. The Y-axis, the percentage of fragments, is the number of DNA fragments measured in a specific length bin, i.e., 0-50 nm, divided by the total number of DNA molecules measured for a sample, and multiplied by 100.

**Figure 1** shows sample AFM images of unirradiated (1a), irradiated to 1000 Gy at 2 (1b-1) and 9.4 cm (1b-2) and 3000 Gy at 2 (1c-1) and 8.9 cm (1c-2) depths. Unirradiated plasmid DNA molecules remained mostly intact, in circular or slightly supercoiled conformation, while plasmids irradiated to 1000 and 3000 Gy yielded progressively more broken DNA molecules in response to dose. Furthermore, **Figures 1b-1, 1b-2** show that irradiation to a dose of 1000 Gy but at two different depths have yielded different amounts of DNA fragmentation. Similarly, as shown in 1c-1 and 1c-2, 3000 Gy at 2 and 8.9 cm yield significantly different amounts of DNA fragmentation, with a more pronounced fragmentation than that for 1000 Gy.

**Figure 2** shows the fragment size distributions at 1000 and 3000 Gy at the two different depths. Both doses yielded greater quantities of unbroken DNA at 2 cm depth than at 9.4 cm depth. For the 1000 Gy irradiated DNA samples, the size distributions of broken fragments at the two depths are largely similar but with somewhat more fragments in the 0-100 nm region at 9.4 cm depth; in the meantime, more than 80% of the DNA remains intact. However, there are marked differences for the 3000 Gy irradiated samples as demonstrated by a pronounced increase in the number of short DNA fragments in the 50-200 nm region at 9.4 cm depth. Furthermore, less DNA remained intact at 9.4 cm than at 2 cm.

From the fragment length distributions of **Figure 2**, we can further calculate the percentage of broken DNA molecules and the average number of DSB per DNA molecule using the formula derived in 24.

**Figure 3:**
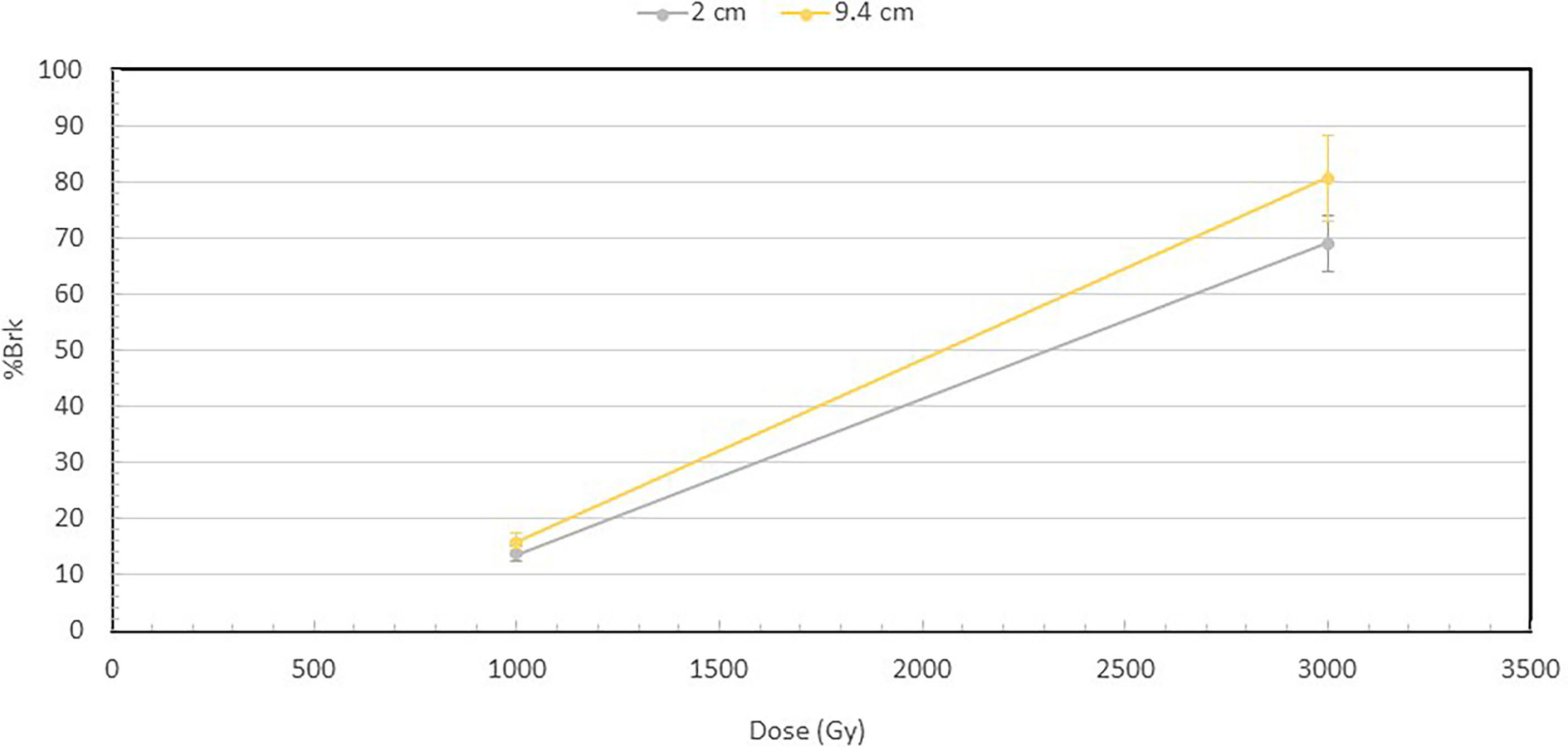
Percentage of broken DNA molecules at the depths of 2 and 9.4 cm for the 1000 and 3000 Gy irradiated DNA. At 1000 Gy, 13.9% of the DNA were broken at 2 cm, while 15.7% at 9.4 cm. At 3000 Gy, 69% of DNA were broken at 2 cm and 80.6% at 9.4 cm. The fraction of broken DNA is the ratio of the number of broken DNA to that of the total number of DNA, which is the sum of both broken DNA and intact DNA. The number of broken DNA is calculated by summing the lengths of the broken DNA fragments and then dividing by the length of an intact DNA.

**Figure 3** shows the percentage of broken DNA at 1000 and 3000 Gy at 2 and 9.4 cm depths. Increase in dose from 1000 to 3000 Gy results in a substantial increase in the fraction of DNA molecules that are broken at both depths. However, a more important observation is that at both 1000 and 3000 Gy, the broken fraction is always higher at 9.4 cm than at 2 cm depth, and the difference increases when the dose is higher.

**Figure 4:**
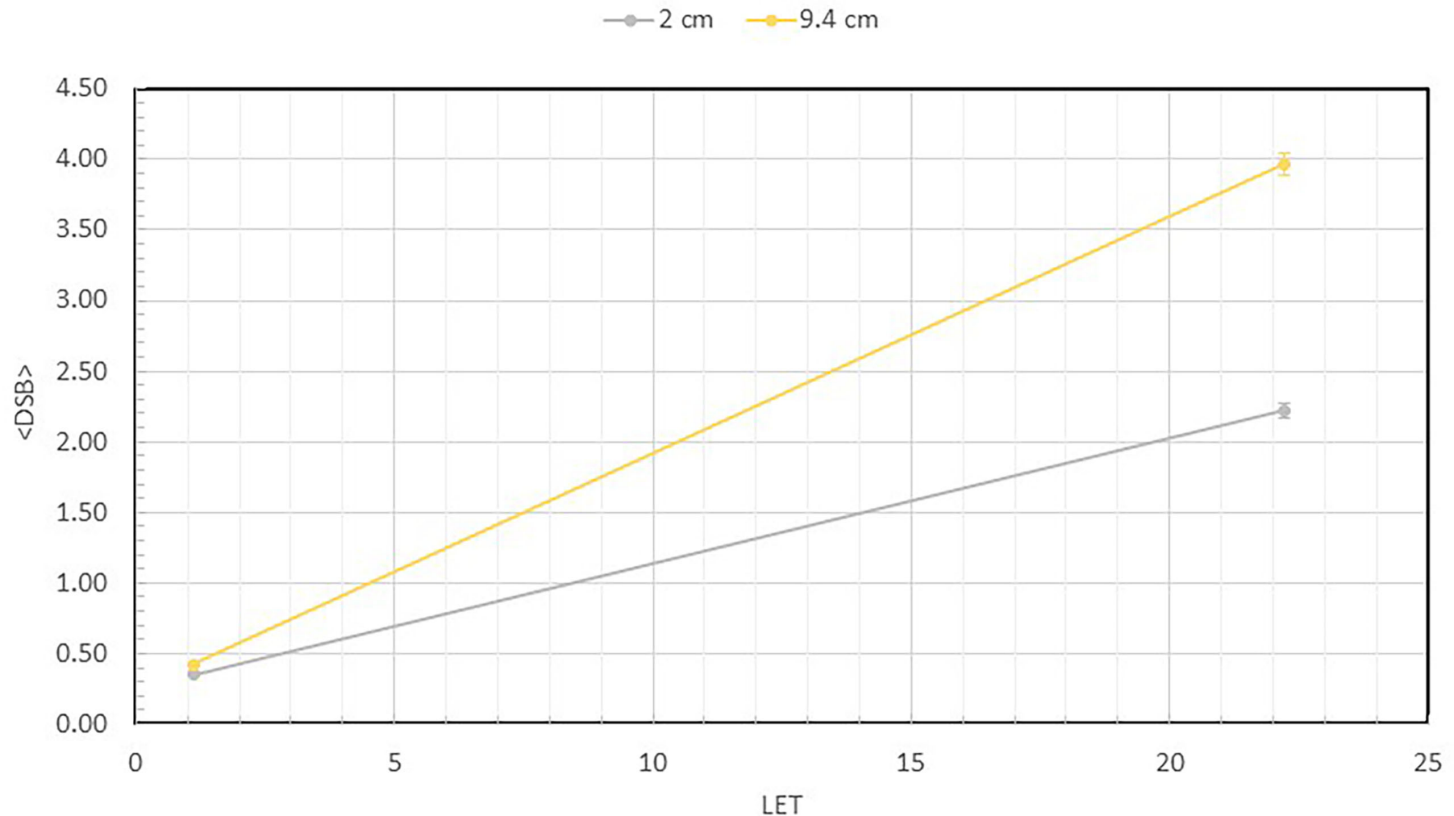
Average number of DSBs per DNA at 2 ad 9.4 cm for both 1000 Gy and 3000 Gy. They are plotted as a function of LET, which shows value of 1.1 keV/µm at 2 cm and 22.6 keV/µm at 9.4 cm depth. This quantity is calculated as the total number of broken DNA fragments divided by the total number of DNA molecules described in **Figure 3** for each sample.

**Figure 4** shows the number of measured DSB per DNA as a function of the LET values at 2 and 9.4 cm depths for both the 1000 and 3000 Gy irradiated DNA samples. At 2 cm depth, the LET is 1.11 keV/µm, but at 9.4 cm depth the LET is substantially higher at 22.6 keV/µm. As shown, the DSB per DNA at both 1000 and 3000 Gy is less than 1 at 2 cm depth. However, at the 9.4 cm depth, the number of DSB per DNA has increased to 2.22 and 3.97, respectively, for the 1000 and 3000 Gy irradiated DNA samples, showing a large dependence on LET.

## Discussion

In this study we investigate critical effects of proton radiation on cells with a focus on potential variation of DNA DSB induction at positions of the Bragg peak. Theoretical calculations have shown a rapid increase in LET along proton beam paths, especially in the Bragg peak region (3). The effects on cell survival have been demonstrated by several investigating groups (5, 27). The increased cell killing capacity of the proton beam in the Bragg peak region, especially towards the end of its range, has significant clinical implications and warrants careful consideration in clinical application of proton irradiation.

The fragmentation distribution profiles shown in **Figure 2** show a dose response. This is consistent with what we have observed with other types of radiation. In our previous work, we observed a difference in DNA fragment distribution profiles resulting from the low-LET photon and high-LET neutron irradiation, which is demonstrated by a profile shift towards shorter DNA fragments by neutron radiation (24). This was interpretated as the result of clustered DNA damage by the higher-LET effects of neutrons. In this work with proton irradiation, we observed a significant increase in the number of short DNA fragments less than 250 nm shown in **Figure 2** for the DNA sample irradiated at the depth of 9.4 cm as compared to the sample irradiated at 2 cm depth at the same dose of 3000 Gy. We interpret this observation to reflect the enhanced LET effect on DNA fragmentation by proton particles with much higher LET at the Bragg peak.

High-LET radiations produce more densely and clustered DNA damage than low-LET radiation. The clustered DNA damage, which produces more short DNA fragments, is difficult to repair by the cells repair mechanism and leads to greater cellular lethality (28, 29). The significantly enhanced short DNA fragments generation at the Bragg peak measured in this study provides firsthand evidence of clustered DNA damage by therapeutic protons in the Bragg peak region and provides a mechanistic support to the previously reported data of enhanced cell killing at the Bragg peak.

From the fragment size distributions in **Figure 2**, we calculated the fraction of broken DNA molecules irradiated at 2 cm and 9.4 cm depth to further illustrate the differential DNA damaging effects of protons along the Bragg peak as shown in **Figure 3**. While there is only a small difference in the number of broken DNAs at the two depths for the 1000 Gy irradiated DNA samples, a substantial difference becomes apparent following exposure of DNA to 3000 Gy irradiation, with 69% of plasmids broken at the 2 cm and 80.6% at the 9.4 cm depth.

To directly quantify the DSB induction, we calculated the average number of DSBs per DNA molecule from the fragment size distribution data using equations from a previous publication (24), and plotted the data as a function of LET. As shown in **Figure 4**, when irradiated to 1000 Gy, the average DSB per DNA molecule are 0.35 and 0.42, respectively, at 2 and 9.4 cm depth. However, when irradiated to 3000 Gy, a substantial increase is seen to reach 2.22 and 3.97, respectively, showing a much greater number of DSB per DNA at the 9.4 cm depth. We calculated the LET of the 110 MeV beam used in our experiments to be 1.1 keV/µm at 2 cm depth and a substantially higher value of 22.6 keV/µm at 9.4 cm depth. The clear dependence of DNA DSBs on LET is a demonstration of the enhanced DNA damaging effect of the higher LET proton particles at the Bragg peak.

Both Monte Carlo simulation of DNA DSB production (30, 31) and our previous work on AFM measurements have provided evidence for the enhanced short DNA fragment production by high-LET radiations. Here, applying the same AFM measurement technique, we determined DNA DSB induction by clinical protons on their path in the plateau and Bragg peak at the same dose, demonstrating an unambiguous capacity of higher LET protons to generate more DSB.

The direct visualization of individual DNA molecules by AFM offers us the possibility of counting and measuring each DNA fragment with an uncertainty of about 20 nm (24) permitting counting and length measurement of DNA fragments individually. This unique ability of the AFM offers insights into radiation induced DNA DSB induction that are difficult to accomplish by other DNA damage assays.

Proton radiotherapy offers a distinct dosimetric advantage over photon radiotherapy in that proton particles traversing tissues have a unique dose deposition pattern exemplified by a largely flat dose-depth correspondence until the end of their paths where the dose increases rapidly to reach a maximum, followed by a subsequent rapid falloff and stopping of all proton particles. Tissue beyond the particle range will receive no dose at all for a complete sparing. The finite range and distal tissue sparing of protons is the primary reason for enthusiastic adoption of proton radiotherapy. However, it was only recently that the potential biological and clinical impact of the higher LET at the Bragg peak, which is inherent to proton and other heavy charged particle beams, began to be explored. The generic RBE value of current clinical proton beams of 1.1 anywhere along a proton beam path was largely based on *in vitro* cell survival data where cell irradiations were performed almost exclusively in the plateau region of the proton depth dose curve (3, 14). Recent investigations have demonstrated a variable RBE with clinical implications.

It is accepted that cell killing correlates to unrepaired DNA DSBs, and the effectiveness of radiation in cell killing is due to its ability to inflict DSB (32–35). High-LET radiations generate more complex DSBs that are more difficult to repair than low-LET radiation (28, 36, 37). Our data on DSB production by clinical proton beams provide direct evidence of variable DSB induction along a proton beam path to support the mechanism underlying the variable RBE for cell survival.

This investigation reports the first experimental evidence of DNA DSB induction variation along the path of a clinical proton beam and shows images of resultant broken DNA strands using a plasmid model system. These data provide support for a mechanistic understanding of the enhanced cell killing at the Bragg peak. The increase in short DNA fragment generation at the Bragg peak and the more densely distributed DNA DSB are consistent with reported RBE values.

## Data availability statement

The raw data supporting the conclusions of this article will be made available by the authors, without undue reservation.

## Author contributions

CF performed AFM imaging of the DNA samples, counted and measured DNA fragment lengths, assisted with data analysis. YC participated in radiation delivery and assisted with data analyses. JG assisted with DNA fragment measurement. TM and YY assisted with radiation delivery and DNA fragment measurement. AD assisted with manuscript preparation. DP conceived and supervised the project execution and wrote the manuscript. All authors contributed to the article and approved the submitted version.

## Conflict of interest

The authors declare that the research was conducted in the absence of any commercial or financial relationships that could be construed as a potential conflict of interest.

## Publisher’s note

All claims expressed in this article are solely those of the authors and do not necessarily represent those of their affiliated organizations, or those of the publisher, the editors and the reviewers. Any product that may be evaluated in this article, or claim that may be made by its manufacturer, is not guaranteed or endorsed by the publisher.
